# Generation of KCL033 clinical grade human embryonic stem cell line

**DOI:** 10.1016/j.scr.2015.12.047

**Published:** 2016-03

**Authors:** Liani Devito, Anastasia Petrova, Victoria Wood, Neli Kadeva, Glenda Cornwell, Stefano Codognotto, Emma Stephenson, Dusko Ilic

**Affiliations:** Stem Cell Laboratories, Division of Women's Health, Faculty of Life Sciences and Medicine, King's College London and Assisted Conception Unit, Guys' Hospital, London, United Kingdom

## Abstract

The KCL033 human embryonic stem cell line was derived from a normal healthy blastocyst donated for research. The ICM was isolated using laser microsurgery and plated on γ-irradiated human foreskin fibroblasts. Both the derivation and cell line propagation were performed in an animal product-free environment and under current Good Manufacturing Practice (cGMP) standards. Pluripotent state and differentiation potential were confirmed by in vitro assays. The line was also validated for sterility and specific and non-specific human pathogens.

## 1 Resource table

**Table:** 

Name of stem cell line	KCL033
Institution	King's College London, London, UK
Derivation team	Neli Kadeva, Victoria Wood, Glenda Cornwell, Stefano Codognotto, Emma Stephenson
Contact person and email	Dusko Ilic, email: dusko.ilic@kcl.ac.uk
Date archived/stock date	Aug 17, 2011
Type of resource	Biological reagent: cell line
Sub-type	Human pluripotent stem cell line
Origin	Human embryo
Key marker expression	Pluripotent stem cell markers: NANOG, OCT4, TRA-1-60, TRA-1-81, alkaline phosphatase (AP) activity
Authentication	Identity and purity of line confirmed
Link to related literature (direct URL links and full references)	1) Jacquet, L., Stephenson, E., Collins, R., Patel, H., Trussler, J., Al-Bedaery, R., Renwick, P., Ogilvie, C., Vaughan, R., Ilic, D., 2013. Strategy for the creation of clinical grade hESC line banks that HLA-match a target population. EMBO Mol. Med. 5 (1), 10–17. doi: 10.1002/emmm.201201973 http://www.ncbi.nlm.nih.gov/pubmed/23161805 2) Canham, A., Van Deusen, A., Brison, D.R., De Sousa, P., Downie, J., Devito, L., Hewitt, Z.A., Ilic, D., Kimber, S.J., Moore, H.D., Murray, H., Kunath, T., 2015. The molecular karyotype of 25 clinical-grade human embryonic stem cells lines. *Sci. Rep.* 5, 17258. doi: 10.1038/srep17258 http://www.ncbi.nlm.nih.gov/pubmed/26607962 3) Ilic, D., Stephenson, E., Wood, V., Jacquet, L., Stevenson, D., Petrova, A., Kadeva, N., Codognotto, S., Patel, H., Semple, M., Cornwell, G., Ogilvie, C., Braude, P., 2012. Derivation and feeder-free propagation of human embryonic stem cells under xeno-free conditions. Cytotherapy. 14 (1), 122–128. doi: 10.3109/14653249.2011.623692 http://www.ncbi.nlm.nih.gov/pubmed/22029654 4) Stephenson, E., Jacquet, L., Miere, C., Wood, V., Kadeva, N., Cornwell, G., Codognotto, S., Dajani, Y., Braude, P., Ilic, D., 2012. Derivation and propagation of human embryonic stem cell lines from frozen embryos in an animal product-free environment. Nat. Protoc. 7 (7), 1366–1381. doi: 10.1038/nprot.2012.080 http://www.ncbi.nlm.nih.gov/pubmed/22722371 5) Devito, L., Petrova, A., Miere, C., Codognottom S., Blakely, N., Lovatt, A., Ogilvie, C., Khalaf, Y., Ilic, D., 2014. Cost-effective master cell bank validation of multiple clinical-grade human pluripotent stem cell lines from a single donor. Stem Cells Transl. Med. 3(10), 1116–1124. doi: 10.5966/sctm.2014-0015 http://www.ncbi.nlm.nih.gov/pubmed/25122690
Information in public databases	KCL033 is a National Institutes of Health (NIH) registered hESC line NIH Registration Number: NIHhESC-14-0267 http://grants.nih.gov/stem_cells/registry/current.htm?id=653
Ethics	The hESC line KCL033 is derived under license from the UK Human Fertilisation and Embryology Authority (research license numbers: R0075 and R0133) and also has local ethical approval (UK National Health Service Research Ethics Committee Reference: 06/Q0702/90). Informed consent was obtained from all subjects and the experiments conformed to the principles set out in the WMA Declaration of Helsinki and the NIH Belmont Report. No financial inducements are offered for donation.

## Resource details

**Table:** 

Consent signed	May 26, 2009
Embryo thawed	Jul 11, 2011
UK stem cell bank deposit approval	Mar 08, 2012 Reference: SCSC12-54
Sex	Female 46, XX
Grade	Clinical
Disease status	Healthy/Unaffected
Karyotype (aCGH)	No copy number changes detected.
SNP array	Gain in regions 5p14.3 and 12p11.21 (Canham et al., 2015)
DNA fingerprint	Allele sizes (in bp) of 16 microsatellite markers specific for chromosomes 13, 18 and 21 (Jacquet et al., 2013)
HLA typing	HLA-A 11,29; B 44,51; Bw 4; C 04,16; DRB1 04,07; DRB4 01; DQB1 02,03 (Jacquet et al., 2013, Canham et al., 2015)
Viability testing	Pass
Mycoplasma	Negative
Sterility	Pass
Pluripotent markers (immunostaining) (Fig. 1)	NANOG, OCT4, TRA-1-60, TRA-1-81
Three germ layers differentiation in vitro (immunostaining) (Fig. 2)	Endoderm: AFP Ectoderm: TUBB3 (tubulin, beta 3 class III) Mesoderm: ACTA2 (actin, alpha 2, smooth muscle)
Sibling lines available	KCL032, KCL034

**Fig. 1:**
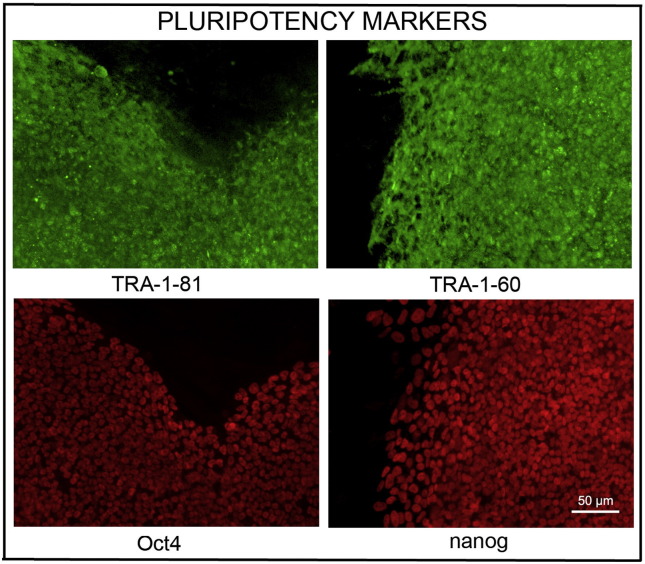
Expression of pluripotency markers. Pluripotency is confirmed by immunostaining (Oct. 4, Nanog, TRA-1-60, TRA-1-81). Scale bar, 50 μm.

**Fig. 2:**
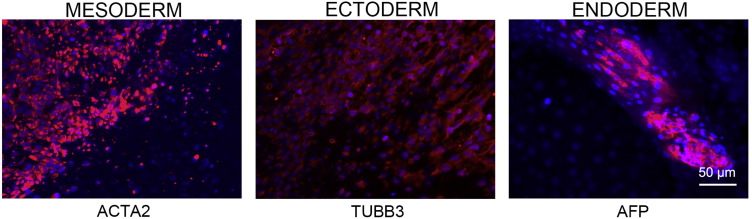
Differentiation of three germ layers in vitro is confirmed by detection of markers: smooth muscle actin (ACTA2, red) for mesoderm, β-III tubulin (TUBB3, red) for ectoderm and α-fetoprotein (AFP, red) for endoderm. Nuclei are visualized with Hoechst 33342 (blue). Scale bar, 50 μm.

We generated KCL033 clinical grade hESC line following protocols, established previously (Ilic et al., 2012, Stephenson et al., 2012), and now adapted to cGMP conditions. The expression of the pluripotency markers was tested after freeze/thaw cycle (Fig. 1). Differentiation potential into three germ layers was verified in vitro (Fig. 2).

Molecular karyotyping identified a novel 2.4 Mb gain on chromosome 5p14.3 and a gain on chromosome 12p11.21, which was also found in KCL040.

The gain on chromosome 5p14.3 containing a single gene, *CDH18* (Cadherin-18), was also present in one of two sibling cell lines, KCL032, but not in KCL034, a third sibling. A duplication of this size has not been reported to date, but its presence in two sibling hESC lines strongly suggests that it was inherited from one of the parents rather than by acquisition during hESC derivation and culture (Canham et al., 2015). The 2498.8 bp gain starts at bp 19086546 and ends at bp 21585311 as referred to Human Genome Build 38.

The gain on chromosome 12p11.21 contains no genes and it has been also reported in at least 14 submissions at Database of Genomic Variants (DGV; http://dgv.tcag.ca), which has collected structural variations in more than 14,000 healthy individuals from worldwide population (MacDonald et al., 2014). Estimated frequency in the human population is 4.70% (Canham et al., 2015).

Validation for sterility and specific and non-specific human pathogens (Devito et al., 2014) conformed that the cells in the Master Bank were sterile, mycoplasma-free, and negative for *Treponema pallidum*, chlamydia, *Neisseria gonorrhoeae*, human immunodeficiency virus-1 and 2 (HIV-1 and -2), human T-lymphotropic virus types-1 and 2 (HTLV-1 and 2), hepatitis A, B and C (HAV, HBV and HCV), human herpes simplex virus HHV-4 (Epstein–Barr virus, EBV), -6, -7, and -8, human cytomegalovirus (hCMV), human parvovirus B19, SV40, JCV, BKV, enterovirus, HAV, HCV, nonspecific viral and other adventitious contaminants.

We also generated research grade of KCL033 line that is adapted to feeder-free conditions.

## Materials and methods

### Consenting process

We distribute Patient Information Sheet (PIS) and consent form to the in vitro fertilization (IVF) patients if they opted to donate to research embryos that were stored for 5 or 10 years. They mail signed consent back to us and that might be months after the PIS and consent were mailed to them. If in meantime new versions of PIS/consent are implemented, we do not send these to the patients or ask them to re-sign; the whole process is done with the version that was given them initially. The PIS/consent documents (FRO-V.6) were created on Dec. 18, 2008. HFEA Code of Practice that was in effect at the time of document creation: Edition 7 — R.4 (http://www.hfea.gov.uk/2999.html). The donor couple signed the consent on May 26, 2009. HFEA Code of Practice that was in effect at the time of donor signature: Edition 7 — R.4. HFEA Code of Practice Edition 7 — R.4 was in effect: 02 Oct. 2008–30 Sep. 2009.

### Embryo culture and micromanipulation

Embryo culture and laser-assisted dissection of inner cell mass (ICM) were carried out as previously described in detail (Ilic et al., 2012, Stephenson et al., 2012). The cellular area containing the ICM was then washed and transferred to plates containing mitotically inactivated human neonatal foreskin fibroblasts (HFF).

### Cell culture

ICM plated on mitotically inactivated HFF were cultured as described (Ilic et al., 2012, Stephenson et al., 2012). TE cells were removed mechanically from the outgrowth (Ilic et al., 2007, Ilic et al., 2010). hES colonies were expanded and cryopreserved at the third passage.

### Viability test

Straws with the earliest frozen passage (p. 2–3) are thawed and new colonies are counted three days later. These colonies are then expanded up to passage 8, at which point cells were part frozen and part subjected to standard battery of tests (pluripotency markers, in vitro and in vivo differentiation capability, genetics, sterility, mycoplasma).

### Pluripotency markers

Pluripotency was assessed with immunostaining for pluripotency markers as described (Ilic et al., 2012, Stephenson et al., 2012).

### Differentiation

Spontaneous differentiation into three germ layers was assessed in vitro and in vivo as described (Petrova et al., 2014, Stephenson et al., 2012).

### Genotyping

DNA was extracted from hES cell cultures using a Chemagen DNA extraction robot according to the manufacturer's instructions. Amplification of polymorphic microsatellite markers was carried out as described (Ilic et al., 2012). Allele sizes were recorded to give a unique fingerprint of each cell line.

### Array comparative genomic hybridization (aCGH)

aCGH was performed as described in detail (Ilic et al., 2012).

### Whole-genome single nucleotide polymorphism (SNP) array

SNP array was performed as described in detail (Canham et al., 2015).

### HLA typing

HLA-A, -B and -DRB1 typing was performed with a PCR sequence-specific oligonucleotide probe (SSOP; Luminex, Austin, TX, USA) hybridization protocol at the certified Clinical Transplantation Laboratory, Guy's and St Thomas' NHS Foundation Trust and Serco Plc. (GSTS) Pathology (Guy's Hospital, London, UK) as described (Jacquet et al., 2013). HLA typing was also performed independently by other group (Canham et al., 2015).

### Validation for sterility and specific and non-specific human pathogens

Validation for sterility and specific and non-specific human pathogens was performed as described (Devito et al., 2014). All validation studies were conducted by SGS Vitrology (Glasgow, U.K., http://www.sgs.com), in compliance with the principles of GMP as set out in Directive 2003/94/EC for medicinal products for human use (Directive 2003/94/EC, 2003) and 91/412/EEC for veterinary medicinal products (Directive 91/412/EEC, 1991).

Sterility testing was performed in accordance with the current requirements of the European Pharmacopoeia, Section 2.6.1 Sterility, U.S. Pharmacopeia, 71. Sterility Tests, and International Conference on Harmonisation Topic Q5D guidelines.

Mycoplasma testing was performed in accordance with the current requirements of the European Pharmacopoeia, Section 2.6.7, Mycoplasmas.

All PCR-based assays used were compliant with the current edition of the European Pharmacopoeia, 2.6.21, Nucleic Acid Amplification Techniques.

## Author disclosure statement

There are no competing financial interests in this study.
